# Supplementary material for: Self-assembly directed one-step synthesis of [4]radialene on Cu(100) surfaces
Source: Nat Commun. 2018 Aug 6;9:3113. doi: 10.1038/s41467-018-05472-2 (PMC6078953; doi:10.1038/s41467-018-05472-2)
Supplement: Supplementary file 1 — Supplementary Information [file 41467_2018_5472_MOESM1_ESM.docx]

Supplementary Information for

Self-assembly directed one-step synthesis of [4]radialene on Cu(100) surfaces

Li *et al.*

**Supplementary Note 1: Comparing the self-assembly and the covalently connected tetramer structures**

Supplementary Figure 1 compares the simulated STM images of the self-assembly (Supplementary Figure 1b) and the covalently connected tetramer (Supplementary Figure 1d) with the experimental observations (Supplementary Figure 1e). It is quite clear that the simulated STM image based on covalent connected structure is in nice agreement with the experimental observation (Supplementary Figure 1e). In contrast, in the simulated STM image based on the self-assembly structure, the monomers appear as elongated protrusions, which resembles the self-assembly hexamers on Au(111).^1^ Moreover, the covalently connected structure is more compact than the self-assembly one. As a consequence, the distance between the centers of the side-protrusions in Fig. S1d is 0.8 nm, in nice agreement with that measured experimentally. On the other hand, the distance between the centers of side-protrusions in Supplementary Figure 1b is slightly larger (0.9 nm). Based on these analysis, we confirm that the observed tetramers on Cu(100) are [4]radialene.


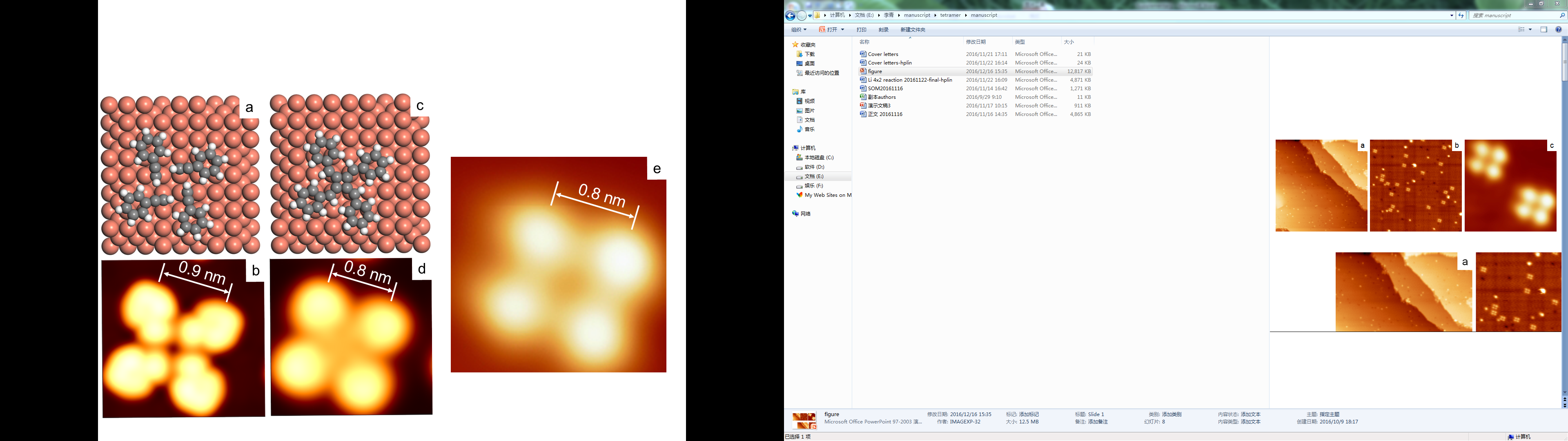


**Supplementary Figure 1 ǀ a,b,** The optimized structure and the corresponding simulated STM image of the non-covalent molecular assembly of four phenylacetylene molecules on the Cu(100) surface. **c,d,** The optimized structure and the corresponding simulated STM image of the covalently bonded tetramer (tetraphenyl[4]radialene) on the Cu(100) surface. Color code: Cu (brown), C (grey) and H (white). **e**, The representative high-resolution STM image of the observed tetramer on Cu(100).

**Supplementary Note 2: Bias dependent images of the tetramers**

The STM images differ significantly under different bias voltages. At higher bias voltages (for example, 1 V in Supplementary Figure 2a), the tip is relatively far away from the target molecule and the molecule is in a strain-free configuration, with four phenyl rings slightly bending up. As a consequence, the STM image exhibits higher protrusions at the phenyl sites, and featureless pattern in the central of the product. At lower bias voltages (for example, 100 meV in Supplementary Figure 2b), the tip is much closer to the target molecule so that the phenyl rings are pushed down to the surface. Therefore the four protrusions of the central four-member carbon ring becomes visible

**
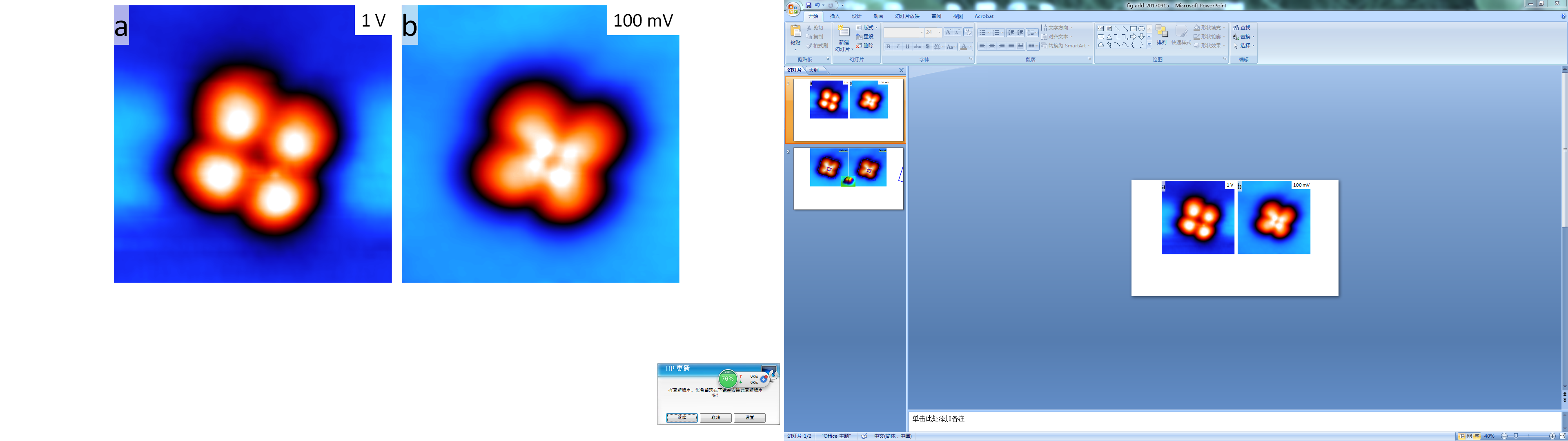
**

**Supplementary Figure 2 ǀ a,b,** The STM images taken with different biases of 1 V and 100 mV, respectively. The image size is 3.1 $\times$ 3.1 nm^2^, taken with the tunneling current of 100 pA for both **a** and **b**.

**Supplementary Note 3: The “tip-molecule” interaction during high-resolution imaging of the single tetramer**

In the imaging of single molecular bond with high resolutions canning probe methods, the “tip-molecule” interaction may play important role. The “tip-molecule” interaction leads to the distortion even displacement of molecules during the scanning. Here, we show two STM images of the same single tetramer molecule, taken simultaneously with forward (right) and backward (left panel) scanning directions (Supplementary Figure 3). We overlaid the height contour plots with STM images, which are used to locate the positions of each carbon atoms of the [4]radialene. As seen, the overall shapes of the [4]radialene are not perfect square and different in the images obtained from forward and backward scanning. Such “tip-molecule” interaction may cause the overestimation of the size of [4]radialene. For clarity, we marked the actual size of the four-member carbon ring with blue squares in both the forward and backward scanning images, as shown in Supplementary Figure 3.


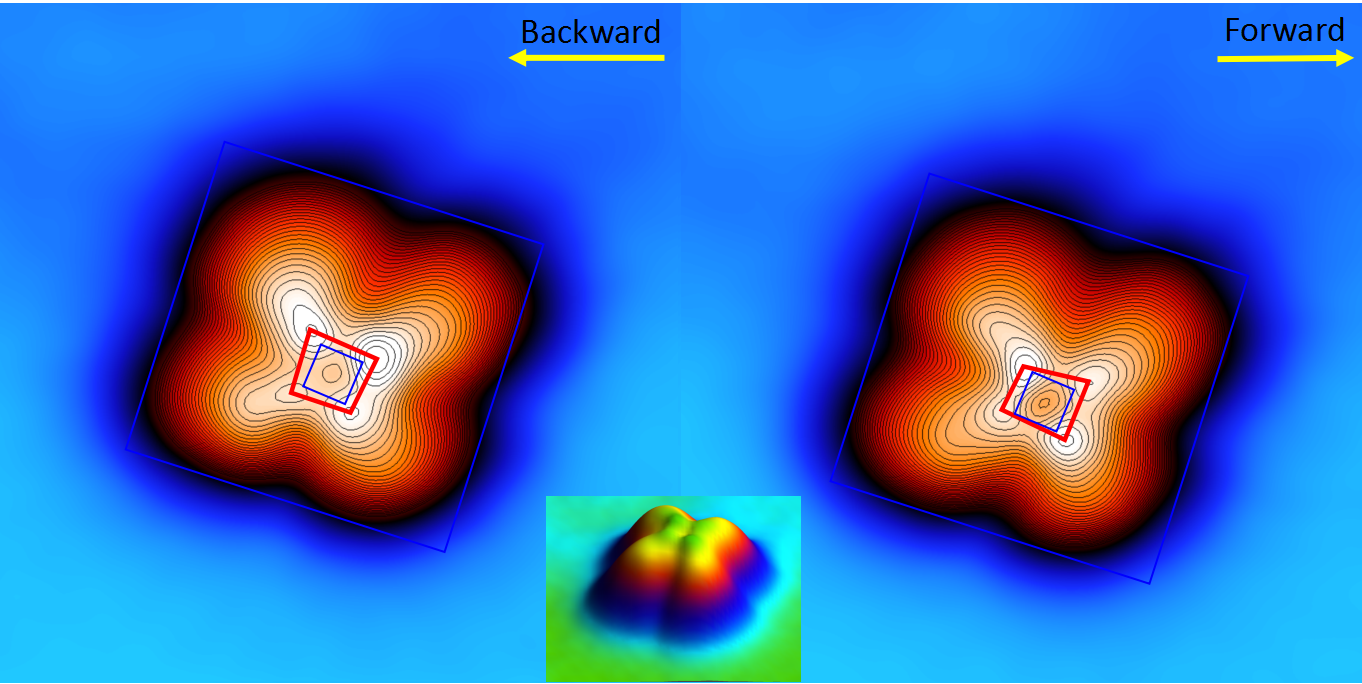


**Supplementary Figure 3 ǀ the tip-crawl effect.** The STM images of a single tetramer product were taken simultaneously with scanning directions of backward (left) and forward (right). The size of images is 3.1 $\times$ 3.1 nm^2^, taken with the bias of 100 mV and the set tunneling current of 100 pA.

**Supplementary Note 4: Candidated tetramer structures based on molecular assembly**

Supplementary Figure 4 gives three candidated tetramer structures, in which the monomers are aggregated via non-covalent interactions. It is very clear that the size of the central square formed by four carbon atoms are much larger than that observed experimentally (1.6 Å, see Fig. 4b in the main manuscript for details). We thus confirm that the observed tetramer are tetraphenyl[4]radialene, rather than the non-covalently bonded structures.


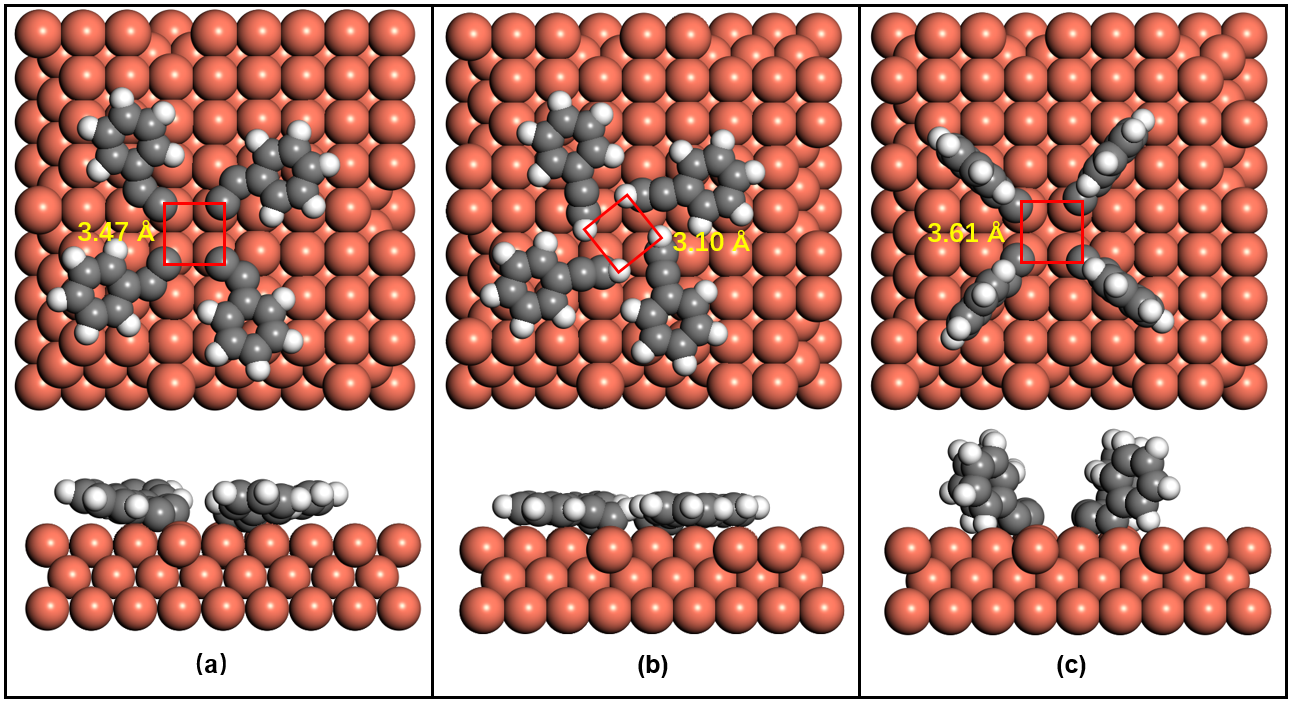


**Supplementary Figure 4 ǀ a-c,** The optimized structures of three candidate non-covalently bonded structure models. The shortest distance between the monomers are 3.47 Å, 3.10 Å and 3.61 Å, respectively. Color code: Cu (brown), C (grey) and H (white).

**Supplementary Note 5: The theoretical studies of the cyclotetramerization mechanism via the non-concerted and concerted intra-molecular hydrogen transfer**

Supplementary Figure 5 gives the reaction pathway of intra-molecular hydrogen transfer, and subsequent C-C bond coupling of two phenylacetylene molecules. The calculated energy barrier is 1.55 eV. The reaction pathway of intra-molecular hydrogen transfer, and subsequent C-C bond coupling of four phenylacetylene molecules to produce the tetraphenyl[4]radialene is shown in Supplementary Figure 6. The calculated energy barrier is 2.59 eV.

**
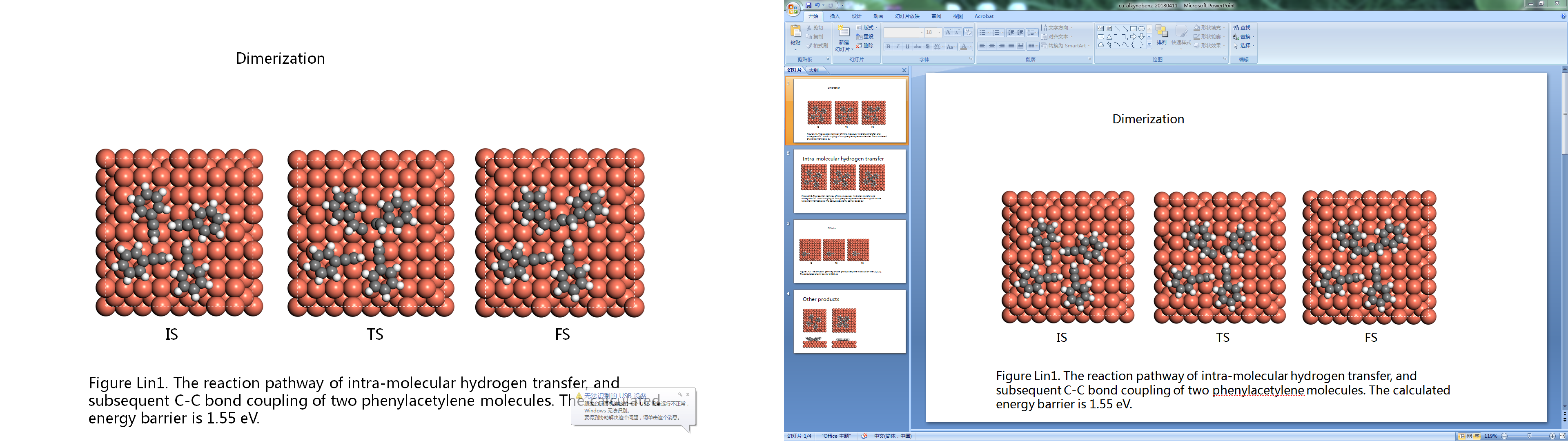
**

**Supplementary Figure 5 ǀ** The reaction pathway of intra-molecular hydrogen transfer, and subsequent C-C bond coupling of two phenylacetylene molecules. The calculated energy barrier is 1.55 eV.

**
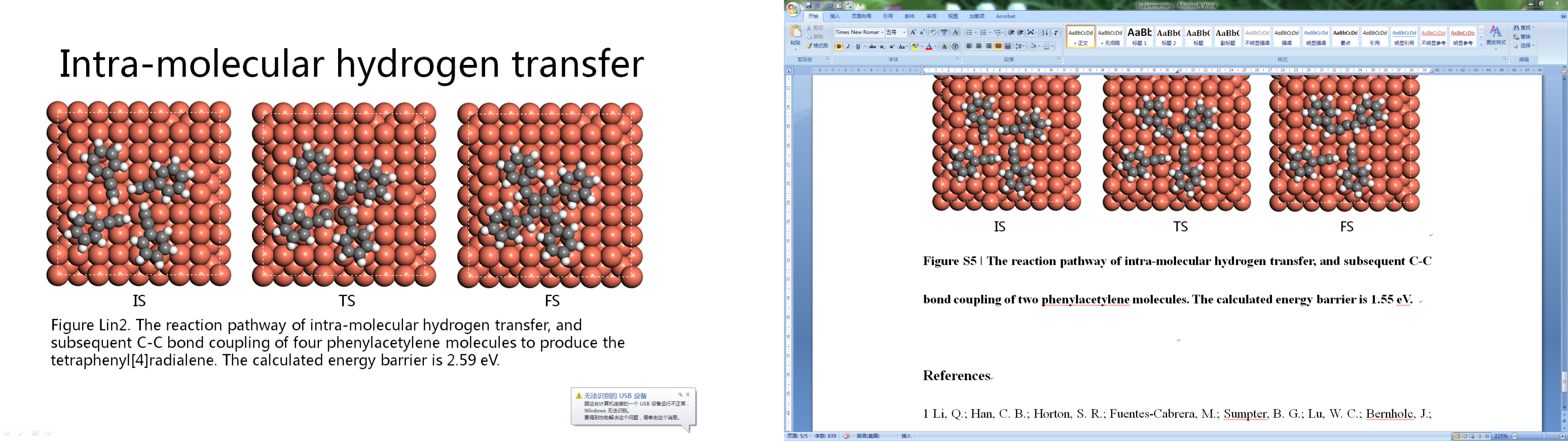
**

**Supplementary Figure 6 ǀ** The reaction pathway of intra-molecular hydrogen transfer, and subsequent C-C bond coupling of four phenylacetylene molecules to produce the tetraphenyl[4]radialene. The calculated energy barrier is 2.59 eV.

**Supplementary Note 6: Noncontact AFM imaging of single [4]radialene**

We reproduced successfully the [4]radialene molecules in different instrument and further characterized by noncontact AFM. Supplementary Figure 7b shows the typical nc-AFM image for a single [4]radialene, acquired with a STM image shown in Supplementary Figure 7a at the same location. At the center of the [4]radialene molecule (marked by yellow dashed rectangle), nc-AFM image shows the sign for two bonds of four-membered ring. Self-assembly and metal-organic complex could not lead to such features.

In addition, one may notice that each phenyl ring of the [4]radialene can only partially be resolved in the nc-AFM image, as shown in Supplementary Figure 7b. It can be explained by the unplanar configurations of the [4]radialenes (similar conclusion can be found in Ref. ^2^). Nc-AFM technique can only images the topmost chemical bonds for an unplanar molecule. In order to make it more clear, the nc-AFM simulations based on out-of-plane state of the [4]radialene (Supplementary Figure 7c) are carried out. As shown in Supplementary Figure 7d, the simulated nc-AFM image only partially resolve the phenyl rings.


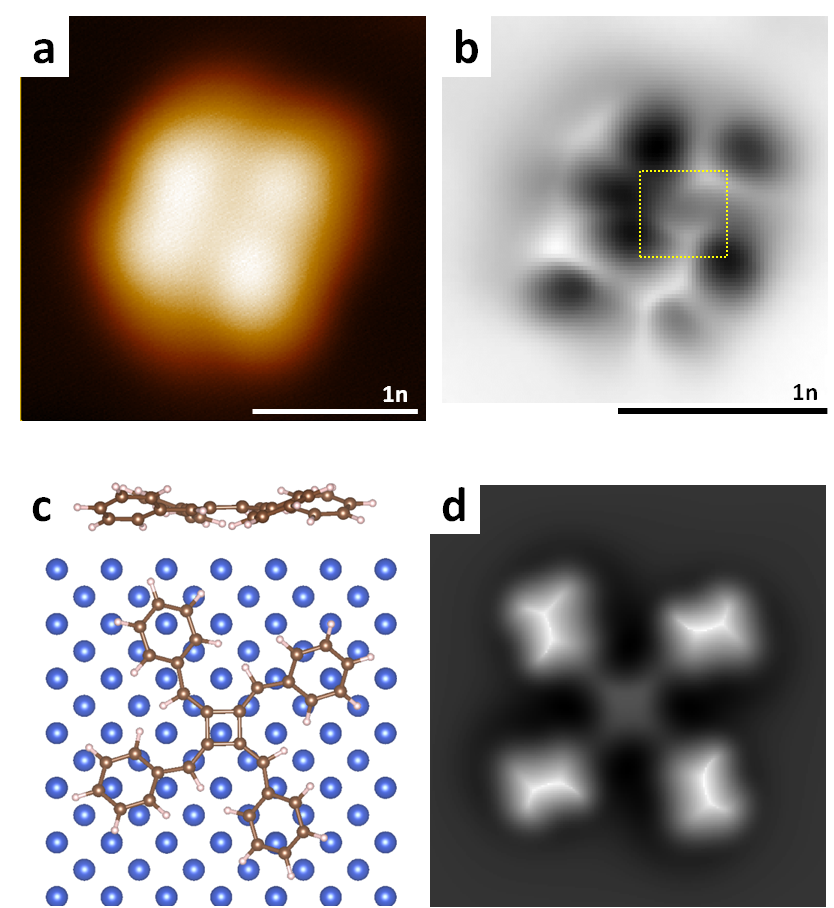


**Supplementary Figure 7.** A typical nc-AFM image **b** for a single [4]radialene, acquired with an STM image at the same location **a. c,** the front and top views of a [4]radialene molecule with unplanar configuration, in which four phenyl-rings tilt slightly out-of-plane and the four-membered ring sinks down. **d,** The simulated nc-AFM image based on the structure shown in **c**.

**Supplementary References**

1. Li, Q. *et al.* Supramolecular self-assembly of π-conjugated hydrocarbons via 2D cooperative CH/π interaction. *ACS Nano* **6**, 566-572 (2012).
2. Shiotari, A. *et al.* Chiral Discrimination and Manipulation of Individual Heptahelicene Molecules on Cu(001) by Noncontact Atomic Force Microscopy. *J. Phys. Chem. C* **122**, 4997-5003 (2018).
